# Heart Rate Variability Is Associated with Exercise Capacity in Patients with Cardiac Syndrome X

**DOI:** 10.1371/journal.pone.0144935

**Published:** 2016-01-26

**Authors:** Dai-Yin Lu, Albert C. Yang, Hao-Min Cheng, Tse-Min Lu, Wen-Chung Yu, Chen-Huan Chen, Shih-Hsien Sung

**Affiliations:** 1 Department of Medicine, Taipei Veterans General Hospital, Taipei, Taiwan; 2 Department of Psychiatry, Taipei Veterans General Hospital, Taipei, Taiwan; 3 Department of Medical Education, Taipei Veterans General Hospital, Taipei, Taiwan; 4 Institute of Clinical Medicine, National Yang-Ming University, Taipei, Taiwan; 5 Department of Medicine, National Yang-Ming University, Taipei, Taiwan; 6 Department of Public Health, National Yang-Ming University, Taipei, Taiwan; 7 Research Center for Adaptive Data Analysis, National Central University, Taoyuan, Taiwan; Children's National Medical Center, Washington, UNITED STATES

## Abstract

Heart rate variability (HRV) reflects the healthiness of autonomic nervous system, which is associated with exercise capacity. We therefore investigated whether HRV could predict the exercise capacity in the adults with cardiac syndrome X (CSX). A total of 238 subjects (57±12 years, 67.8% men), who were diagnosed as CSX by the positive exercise stress test and nearly normal coronary angiogram were enrolled. Power spectrum from the 24-hour recording of heart rate was analyzed in frequency domain using total power (TP) and spectral components of the very low frequency (VLF), low frequency (LF) and high frequency (HF) ranges. Among the study population, 129 subjects with impaired exercise capacity during the treadmill test had significantly lower HRV indices than those with preserved exercise capacity (≥90% of the age predicted maximal heart rate). After accounting for age, sex, and baseline SBP and heart rate, VLF (odds ratio per 1SD and 95% CI: 2.02, 1.19–3.42), LF (1.67, 1.10–2.55), and TP (1.82, 1.17–2.83) remained significantly associated with preserved exercise capacity. In addition, increased HRV indices were also associated with increased exercise duration, rate-pressure product, and heart rate recovery, independent of age, body mass index, and baseline SBP and heart rate. In subgroup analysis, HRV indices demonstrated similar predictive values related to exercise capacity across various subpopulations, especially in the young. In patients with CSX, HRV was independently associated with exercise capacity, especially in young subjects. The healthiness of autonomic nervous system may have a role in modulating the exercise capacity in patients with CSX.

## Introduction

Cardiac syndrome X (CSX), a clinical condition characterized by exertional angina, exercise induced myocardial ischemia, and normal coronary angiogram [1], is associated with coronary microvascular insufficiency [2]. It has been suggested that automonic dysfunction may contribute to the increased vasomotion of the pre-arteriolar coronary vessels in CSX [3], as demonstrated in studies employing myocardial 123I-metaiodobenzylguanidine scintigraphy [4] or assessing baroreflex sensitivity [5] and heart rate variability (HRV) [5, 6]. In addition, the parasympathetic withdrawal quantitated by HRV is associated with reduced coronary flow reserve, antedating episodes of dynamic myocardial ischemia [6]. CSX patients may have a good long-term survival. However, the attendant morbidity is not negligible [7], since the symptomatic patients may confront significant limitation of physical activities due to chest pain.

Spectral analysis of HRV has been used widely as a non-invasive technique for examining sympathetic and parasympathetic nervous outflows to the heart, which have been associated with the presence and the prognosis of cardiac disorders, including coronary artery disease (CAD), fatal arrhythmia, and heart failure [8–10]. It has been shown that regular physical activity or exercise training may reverse the autonomic neural remodeling in subjects with CAD, myocardial infarction, or heart failure [11–13] with resultant improvement in the clinical outcomes [14]. However, HRV indices were not always related to physical activity or exercise tolerability in patient [15] or in the general population [16]. It remains unknown whether the cardiac autonomic function modulates exercise capacity in CSX patients. In the present study, we therefore investigated the association between HRV and exercise capacity in the patients with CSX.

## Methods

### Study population

The study population was drawn from an intramural registry of **Ta**ipei Vete**r**ans **Ge**neral Hospi**t**al (TARGET registry), conducted to enroll patients referred for non-invasive studies due to the clinical impression of CAD. In addition to treadmill exercise test, ambulatory ECG monitoring, echocardiography, the medical history, the findings of physical examinations, and biochemical examinations were also prospective logged in a web-based electronic medical recording system. Estimated glomerular filtration rate (eGFR) was calculated with the published formula for Chinese [17]. Patients with stable angina, who have received both exercise treadmill test (CASE T2100, General Electric Company, USA) and 24-hour ambulatory ECG monitoring (Medilog FD4, Oxford Instruments, UK) before coronary angiogram, were eligible for this study.

From 2009 to 2013, a total of 238 subjects who have sinus rhythm, myocardial ischemia documented by the exercise stress test, and consequent normal or nearly normal coronary angiogram on the basis of the visual inspection by two experienced cardiologists were enrolled to the present analysis. Patients with significant valvular heart disease, heart failure, cardiomyopathy, atrial arrhythmia, sick sinus syndrome, pacing rhythm, pulmonary disease, stroke and other neurological disorders, or musculoskeletal pain has been excluded. The investigation conformed to the principles outlined in the Declaration of Helsinki, and the institutional review board of Taipei Veterans General Hospital approved the study. The participants provided their written informed consent to participate in this study, and the institutional review board approved this consent procedure.

### Exercise stress test

Participants were refrained from smoking or drinking beverages containing caffeine or alcohol for 24 hours before exercise stress test and 24-hour ambulatory ECG monitoring. All patients would undergo symptom-limited exercise testing using a Bruce protocol. Twelve-lead ECG was obtained throughout the test, and exercise blood pressures were measured at baseline, during the last minute of each 3-minute stage, at the moment of maximum effort, and at 1 minute after the test with the arm relaxed at the side without holding on to the side bar of the treadmill, using an incorporated automatic BP monitor. (CASE T2100, General Electric Company, USA) A physician and two experienced technicians conducted the exercise test, and patients were encouraged continuing exercise if there was no severe electrocardiographic ischemia or unstable hemodynamics. The maximal systolic blood pressure (SBP) was defined as the highest value achieved during the test. Total exercise duration was documented and the rate-pressure product was the product of maximal achieved SBP and heart rate during exercise. Heart rate recovery is defined as the difference in heart rate between peak exercise and 1 minute later [18]. Patients who achieved more than 90% of the age predicted maximal heart rate were defined to have preserved exercise capacity, while the others had impaired exercise capacity.

### Measures of Heart Rate Variability

A 24-hour ECG signal provided by the use of 3-channel digital recorders (Medilog FD4, Oxford Instruments, UK) was stored for off-line analyzing. The digitized ECG signals were processed and analyzed using open source HRV algorithms [19]. Briefly, the time series of R-R intervals was filtered to remove ectopic beats (such as supraventricular or ventricular ectopic beats), missing or noisy segments by linear interpolation from the surrounding signal. Only subjects with over 18 hours of good quality of normal-to-normal interbeat intervals were entered the subsequent HRV analyses. For each R-R time series, the tachogram was re-sampled at 4 Hz (http://ecg.mit.edu/dbag/tach-1.htm) to generate a uniformly spaced time series.

The computational algorithms for spectral HRV indices employed in this study are available publicly at www.physionet.org. Spectral HRV measures [20] were calculated using Fast Fourier transform with Welch window and include high-frequency power (HF; 0.15–0.40 Hz), low-frequency power (LF; 0.04–0.15 Hz), and very-low-frequency power (VLF; 0.003–0.04 Hz). The spectral HRV indices were calculated for each non-overlapping 5 minutes window of R-R time series and the average of VLF, LF, and HF spectral power was obtained to represent the overall HRV measures for a subject. A natural logarithmic transformation was used to normalize the distribution of the spectral HRV measures. LF power is suggested to be modulated by both sympathetic and parasympathetic activities, whereas HF power is primarily modulated by parasympathetic activity [21, 22]. The LF/HF ratio was computed as a measure of the sympathovagal balance toward sympathetic activity [20, 23]. The physiological mechanism underlying VLF power is disputed but has been suggested to be partially mediated by the renin-angiotensin-aldosterone system [20, 24, 25].

### Statistical analysis

Means, standard deviations, and percentages were used to describe the characteristics of participants in the study. Student’s t-test and Chi-square test were used for comparisons between subjects with and without achieving more than 90% of the age predicted maximal heart rate. Uni- and multi-variate linear regression analyses were performed to examine the associations of HRV indices with exercise capacity, in terms of exercise duration and achieved METs. The association of each HRV index with exercise capacity was separately evaluated by multi-variate logistic regression analyses with adjustments for age, gender, baseline SBP and heart rate, and use of β-blockers and renin-angiotensin system blockers. Forward stepwise multiple logistic regression analyses were used to compare the predictive values of HRV indices after accounting for age, gender, baseline SBP and heart rate. Subgroup analysis was performed stratified by age of 60 year-old, gender, and presence of co-morbidities. A p value < 0.05 was considered statistically significant, and all statistical analyses were carried out using SPSS 17.0 (SPSS Inc., Chicago, IL, USA).

## Results

Among the total of 238 patents (aged 57.0 ± 12.4 years, 67.8% men), 109 subjects (45.8% of total population) achieved more than 90% of the maximal age predicted heart rate during exercise stress test. Comparing to those without such achievement, subjects with good exercise capacity were characterized by younger age and less hypertension. (Table 1) Gender distribution, body mass index, diabetic prevalence, renal function, lipid profiles, and fasting blood sugar were similar between these 2 groups. In addition, patients with good exercise capacity were less likely to take non-dihydropiridine calcium channel blockers (CCBs) and renin-angiotensin system blockade. Otherwise, the prescriptions of β-blockers, α-blockers, and Statin between 2 groups were the same.

**Table 1:** Baseline characteristics of the study population.

	Impaired exercise capacity, n = 129	Preserved exercise capacity, n = 109	*p*-value
Age, years	59.97 ± 11.18	53.55 ± 12.95	<0.001
Male gender, n (%)	82(63.6)	80(73.4)	0.139
BMI, kg/m^2^	25.40 ± 3.92	24.77 ± 2.96	0.191
**Co-morbidities**			
Hypertension, n (%)	67 (51.9)	34 (31.2)	0.002
Diabetes, n (%)	28 (21.7)	20 (18.3)	0.631
**Biochemistry profiles**			
eGFR, ml/min/1.73 m^2^	84.67 ± 30.44	88.48 ± 26.17	0.328
Cholesterol, mg/dL	180.42 ± 30.56	176.69 ± 32.48	0.388
HDL-cholesterol, mg/dL	52.31 ± 16.05	49.67 ± 13.37	0.217
LDL-cholesterol, mg/dL	111.69 ± 30.55	109.60 ± 28.38	0.627
Triglyceride, mg/dL	111.29 ± 74.97	118.97 ± 76.51	0.455
Fasting Glucose, mg/dL	102.22 ± 22.90	99.42 ± 24.42	0.381
**Prescribed medications**			
Dihydropyridine CCB, n (%)	40 (31)	10 (9.2)	<0.001
β-blockers, n (%)	38 (29.5)	21 (19.3)	0.096
α-blockers, n (%)	4 (3.1)	1 (0.9)	0.474
RAS blockers, n (%)	45 (34.9)	23 (21.1)	0.028
Statin n (%)	34 (26.4)	23 (21.1)	0.427

BMI = body mass index; CCB = calcium channel blocker; eGFR = estimated glomerular filtration rate; HDL = high density lipoprotein; LDL = low density lipoprotein; METs = metabolic equivalents; RAS = renin-angiotensin system.

No doubt patients with good exercise capacity experienced longer exercise test duration, and they achieved higher METs and Bruce treadmill test stage. (Table 2) The baseline SBP and pulse pressure (PP) were lower and heart rate was higher in patients with good exercise capacity. But the diastolic blood pressure and mean arterial blood pressure were similar. Regarding HRV indices, VLF power, LF power, and total power (TP), but not LF/HF were higher in the patients with good exercise capacity. However, there was a borderline significantly difference in HF power between the two groups.

**Table 2:** Characteristics of the exercise stress testing and heart rate variability in patients with impaired and preserved exercise capacity.

	Impaired exercise capacity, n = 129	Preserved exercise capacity, n = 109	*p*-value
**Exercise stress test**			
Total exercise duration, s	475.15 ± 165.28	535.32 ± 150.77	0.004
Achieved METs	10.62 ± 2.64	11.53 ± 2.35	0.006
Achieved Bruce Treadmill stage	3.21 ± 0.96	3.53 ± 0.85	0.007
**Baseline blood pressure and heart rate**			
SBP, mmHg	127.05 ± 19.49	121.86 ± 18.07	0.036
DBP, mmHg	76.47 ± 13.11	76.81 ± 11.45	0.836
MAP, mmHg	93.33 ± 13.83	91.83 ± 12.46	0.382
PP, mmHg	50.57 ± 14.97	45.06 ± 13.61	0.003
Heart rate, beats/min	73.40 ± 13.45	82.93 ± 16.32	<0.001
**Maximal achieved blood pressure and heart rate**			
SBP, mmHg	154.86 ± 30.79	149.57 ± 28.53	0.173
DBP, mmHg	74.06 ± 15.32	75.10 ± 14.56	0.594
MAP, mmHg	100.99 ± 17.78	99.92 ± 15.90	0.619
PP, mmHg	80.80 ± 28.55	74.47 ± 26.84	0.081
Heart rate, beats/min	140.12 ± 17.69	181.16 ± 71.17	<0.001
**Increase of blood pressure and heart rate**			
SBP change, mmHg	30.95 ± 30.50	36.92 ± 22.62	0.093
Heart rate change, beats/min	62.32 ± 20.88	97.79 ± 70.15	<0.001
Rate-pressure product, bpm*mmHg	21804.88 ± 5476.91	25990.32 ± 4719.74	<0.001
**Heart rate variability**			
Mean heart rate, beats/min	77.73 ± 13.52	83.37 ± 14.70	0.002
*VLF, ms^2^	3498.19 ± 2.80	4817.45 ± 1.81	0.004
*LF, ms^2^	871.31 ± 3.00	1261.43 ± 2.29	0.005
*HF, ms^2^	595.86 ± 3.67	804.32 ± 2.97	0.052
*TP, ms^2^	5324.10 ± 2.53	7186.80 ± 1.88	0.004
LF/HF	1.82 ± 1.26	1.90 ± 1.21	0.612

*Geometric mean and standard deviation.

Rate-pressure product = maximal heart rate * maximal SBP.

BMI = body mass index; DBP = diastolic blood pressure; eGFR = estimated glomerular filtration rate; HF = high frequency component of the heart rate variability power spectrum; LF = low frequency component of heart rate variability power spectrum; MAP = mean blood pressure; METs = metabolic equivalents; RAS = renin-angiotensin system; SBP = systolic blood pressure; TP = total power of the heart rate variability power spectrum; VLF = very low frequency of the heart rate variability power spectrum.

### Predictors of achieving 90% predicted heart rate

Age, heart rate, SBP, PP as well as HRV indices, including VLF power, LF power and TP were all predictors of achieving 90% maximal predicted heart rate. (Table 3) After accounting for age, gender, baseline SBP and heart rate in multivariate logistic regression analysis, VLF power, LF power, and TP, but not HF power significantly predicted the achievement of 90% predicted heart rate. (Table 4) With further adjustments for the use of β-blockers and renin-angiotensin system blockade, VLF, LF power and TP power remained associated with exercise capacity.

**Table 3:** Determinants of achieving more than 90% of the age predicted maximal heart rate during exercise stress test: univariate logistic regression analysis.

Variable	Odd ratio (95% CI)	P value
Age, 1SD = 12.40 year	0.573 (0.431–0.762)	<0.001
Baseline heart rate, 1SD = 14.38 bpm	1.512 (1.150–1.987)	0.003
Baseline SBP, 1SD = 18.99 mmHg	0.756 (0.580–0.984)	0.037
Baseline PP, 1SD = 14.60 mmHg	0.673 (0.513–0.883)	0.004
**Heart rate variability indices**		
lnVLF, 1SD = 0.87 ms^2^	1.722 (1.192–2.486)	0.004
lnLF, 1SD = 1.00 ms^2^	1.499 (1.125–1.996)	0.006
lnHF, 1SD = 1.22 ms^2^	1.295 (0.996–1.683)	0.053
lnTP, 1SD = 0.82 ms^2^	1.554 (1.144–2.110)	0.005
LF/HF, 1SD = 1.23	1.068 (0.828–1.379)	0.611

CI = confidence interval; HF = high frequency component of heart rate variability power spectrum; LF = very low frequency component of heart rate variability power spectrum; PP = pulse pressure; SBP = systolic blood pressure; SD = standard deviation; TP = total power of heart rate variability power spectrum; VLF = very low frequency component of heart rate variability power spectrum.

**Table 4:** Determinants of achieving more than 90% of the age predicted maximal heart rate during exercise stress test: multi-variate logistic regression analysis*.

Variable	Model 1		Model 2	
	OR (95% CI)	P value	OR (95% CI)	P value
lnVLF, 1SD 0.87 ms^2^	2.016 (1.189–3.420)	0.009	1.989 (1.154–3.427)	0.013
lnLF, 1SD 1.00 ms^2^	1.673 (1.096–2.552)	0.017	1.639 (1.072–2.506)	0.022
lnHF, 1SD 1.22 ms^2^	1.419 (1.000–2.015)	0.050	1.420 (0.996–2.025)	0.053
lnTP, 1SD 0.82 ms^2^	1.820 (1.170–2.830)	0.008	1.792 (1.143–2.809)	0.011

*each index of heart rate variability was evaluated separately

Model 1: accounting for age, sex, baseline systolic blood pressure and heart rate

Model 2: Model 1 + use of β-blockers and renin-angiotensin system blockers

CI = confidence interval; HF = high frequency component of heart rate variability power spectrum; LF = very low frequency component of heart rate variability power spectrum; OR = odds ratio; TP = total power; VLF = very low frequency component of heart rate variability power spectrum.

### Determinants of exercise duration

While age, body mass index, and baseline SBP, PP and HR significantly correlated with total exercise duration, rate-pressure product during treadmill test, and heart rate recovery, VLF, LF and TP were also related to those measures. After accounting for age, body mass index, and baseline SBP and HR, VLF, LF and TP remained associated with total exercise duration and rate-pressure product. (Table 5) In addition, VLF, LF, HF and TP were associated with heart rate recovery in multivariate linear regression analysis. (Table 5)

**Table 5:** Correlates of exercise duration, achieved metabolic equivalent, and rate- pressure product with heart rate variability*^†^.

	Exercise duration		Rate-Pressure product		Heart rate recovery	
	Standardized coefficient	P value	Standardized coefficient	P value	Standardized coefficient	P value
lnVLF, ms^2^	0.252	<0.001	0.261	<0.001	0.246	0.001
lnLF, ms^2^	0.151	0.035	0.122	0.049	0.244	0.002
lnHF, ms^2^	0.032	0.649	0.057	0.361	0.201	0.008
lnTP, ms^2^	0.193	0.006	0.186	0.003	0.252	0.001
LF/HF	0.153	0.029	0.015	0.809	-0.075	0.322

*accounting for age, body mass index, baseline systolic blood pressure and heart rate.

^†^accounting for age, body mass index, baseline systolic blood pressure and heart rate.

HF = high frequency component of heart rate variability power spectrum; LF = very low frequency component of heart rate variability power spectrum; TP = total power of heart rate variability power spectrum; VLF = very low frequency component of heart rate variability power spectrum.

### Subgroup analysis in predicting the achievement of 90% predicted heart rate

**Fig 1:**
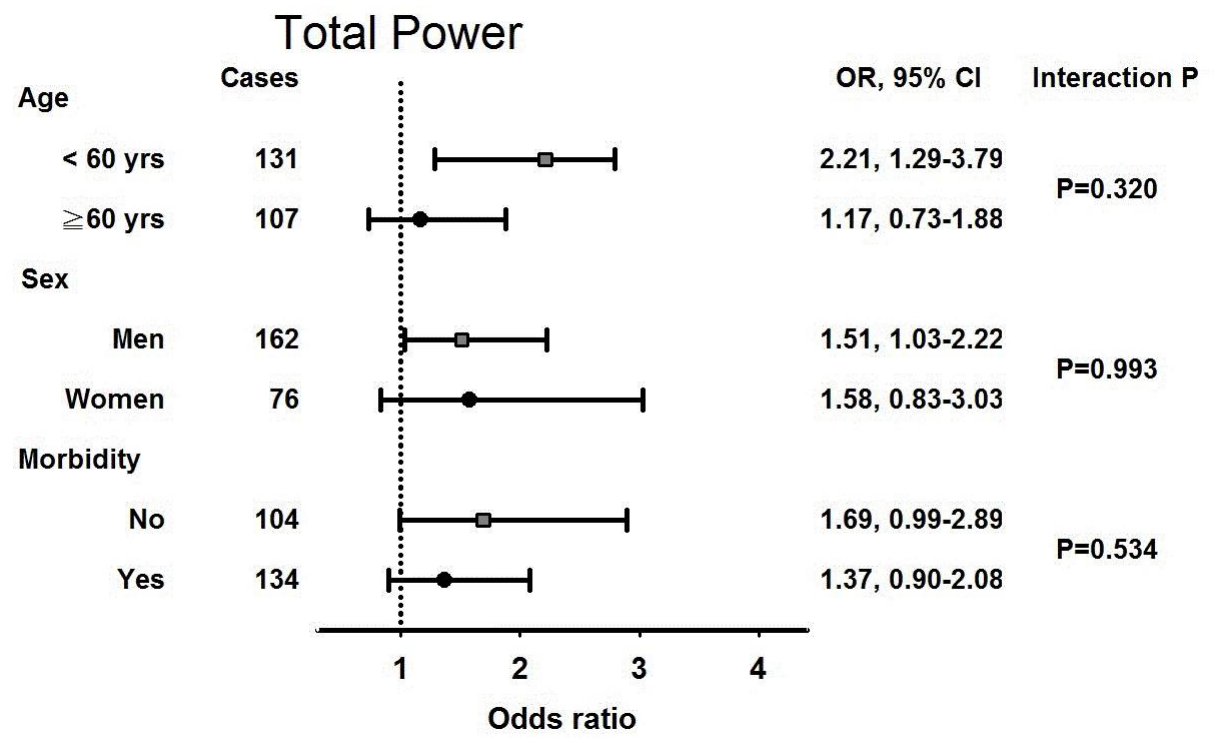
Odds ratio (OR) and 95% confidence interval (CI) per increment of 1 standard deviation of total power heart rate variability power spectrum for preserved exercise capacity in the younger (< 60 years) and the older (≥ 60 years) subjects; women and men; subjects with and without hypertension or diabetes, after accounting for age.

In subgroup analysis, high total power HRV was significantly associated with the achievement of 90% predicted heart rate in in younger rather than older subjects, and in male rather than female subjects, after accounting for age. (Fig 1) However, there was no substantial interaction across the subgroups, regarding age, gender, and co-morbidities. In addition, VLF, LF and HF also demonstrated similar clinical correlates with exercise capacity in subgroup analyses. (S1–S3 Figs)

## Discussion

The present study demonstrated that exercise intolerance indeed prevailed in patients with CSX, while 54.2% of the study population had ischemia-limited impaired exercise capacity. In addition, subjects with impaired exercise tolerance were characterized by higher SBP and PP, and lower HRV indices. These HRV indices were not only related to exercise duration and rate-pressure product, but also independent predictors of ischemia associated exercise intolerance in CSX patients. Furthermore, the relations of HRV to exercise tolerability consistently existed in various subpopulations of the younger and older subjects, men and women, and subjects with and without co-morbidities.

### Heart rate variability and exercise capacity

It has been known that autonomic activity may affect the endurance of exercise, because parasympathetic withdraw and a sympathetic augmentation [26] during dynamic training may increase heart rate, reduce the muscle blood flow and thus oxygen supply. Accordingly, the cardiac autonomic activity could correlate with the exercise tolerability, as assessed by the anaerobic threshold or the peak oxygen uptake [27]. In the community-based Cardiovascular Health Study of 985 adults, physical activity of walking distance or walking pace was both cross-sectionally and longitudinally associated with higher HRV [28]. Over 5 years, those who increased their walking pace or walking distance had more favorable HRV indices in comparison with the others. Not only in general populations but also in athletes and patients with existed cardiovascular disease, exercise training program may propose favorable impacts on cardiac autonomic activity with increasing HRV [29, 30].

### Heart rate variability and cardiac syndrome X

An autonomic imbalance has been observed in subjects with CSX, involving the dynamic variations in the vasomotor tone of coronary microcirculations and thereafter precipitating the transient ischemic episodes [6, 31]. While some studies demonstrated that patents with CSX had lower HRV comparing to healthy controls [5, 32], Gulli et al. further suggested parasympathetic impairment rather than sympathetic over-excitation in the pathogenesis of CSX [33]. Moreover, Lee et al. indicated that the vagal withdraw was involved in the pathogenesis of dynamic myocardial ischemia, because HF power and root mean square of the successive differences (RMSSD) were significantly reduced before ischemic episodes [6]. Accordingly, cardiac autonomic activity may be associated with ischemia-limited exercise in patients with CSX.

In the present study, we demonstrated an agreeing result that patients with CSX who achieved 90% maximal predicted heart rate during treadmill exercise test had higher HRV comparing to those who did not. The association was independent of age, sex, baseline blood pressure and heart rate, and uses of antihypertensive agents. HRV was also independently correlated with exercise duration and rate pressure product, indicating that cardiac autonomic activity was essential to the cardiopulmonary function in patients with CSX [34].

### Heart rate variability and exercise capacity in the elderly with cardiac syndrome X

Measures of HRV decrease linearly with age, indicating an age-related reduction in the responsiveness of autonomic activity [35]. Levy et al. reported that older subjects might have less parasympathetic withdrawal than the young subjects during peak exercise [36]. In the present study, we showed that all HRV indices were more predictive in predicting ischemia-limited exercise intolerance in patients younger than 60 years (Fig 1 and S1–S3 Figs). In contrast, the correlations between HRV indices and exercise capacity were similar in men and women, and in patients with and without hypertension or diabetes.

### Study limitation

There are several limitations in the present observational study. First, good exercise capacity and exercise training are usually related to a lower resting heart rate, which is not observed in this study. But subjects with poor exercise capacity in this study tend to take more beta-blockers, which would confound the mean and resting heart rate. Although we have done multiple logistic regression analyses, accounting for the confounders and medications, omitted variable bias remained. Second, we have excluded subjects with documented pulmonary disease. However, some patients might have limited exercise capacity due to abnormal pulmonary function, which was not examined in the present study. In addition, the participants may not be compliant to the study protocol; even we did have experienced technicians encouraging them to achieve their predicted peak heart rate. Third, the exercise capacity is determined not only cardiopulmonary function, but also non-cardiac factors, including mental stress. Although the coefficients of the associations between HRV and exercise duration, rate-pressure product or heart rate recovery in multiple linear models were relatively small, it still suggested a consistent relation of HRV and exercise. Finally, a causal relationship between autonomic activity and exercise intolerance in patients with CSX could not be established in the present cross-sectional study. A longitudinal study may be warranted.

## Conclusions

In patients with CSX, a reduction in cardiac autonomic activity is associated with impaired exercise capacity. Since anaerobic exercise training may improve autonomic activity, patients with CSX may be encouraged to exercise regularly to augment the favorable autonomic function and subsequently improve their quality of life.

## Supporting Information

S1 Fig Odds ratio (OR) and 95% confidence interval (CI) per increment of 1 standard deviation of very low frequency power of heart rate variability power spectrum for preserved exercise capacity in the younger (< 60 years) and the older (≥ 60 years) subjects; women and men; subjects with and without hypertension or diabetes, after accounting for age. (TIF)

S2 Fig Odds ratio (OR) and 95% confidence interval (CI) per increment of 1 standard deviation of low frequency power of heart rate variability power spectrum for preserved exercise capacity in the younger (< 60 years) and the older (≥ 60 years) subjects; women and men; subjects with and without hypertension or diabetes, after accounting for age. (TIF)

S3 Fig Odds ratio (OR) and 95% confidence interval (CI) per increment of 1 standard deviation of high frequency power of heart rate variability power spectrum for preserved exercise capacity in the younger (< 60 years) and the older (≥ 60 years) subjects; women and men; subjects with and without hypertension or diabetes, after accounting for age (TIF)
