# Supplementary figures and images for: Heart Rate Variability Is Associated with Exercise Capacity in Patients with Cardiac Syndrome X
Source: PLoS One. 2016 Jan 26;11(1):e0144935. doi: 10.1371/journal.pone.0144935 (PMC4727925; doi:10.1371/journal.pone.0144935)

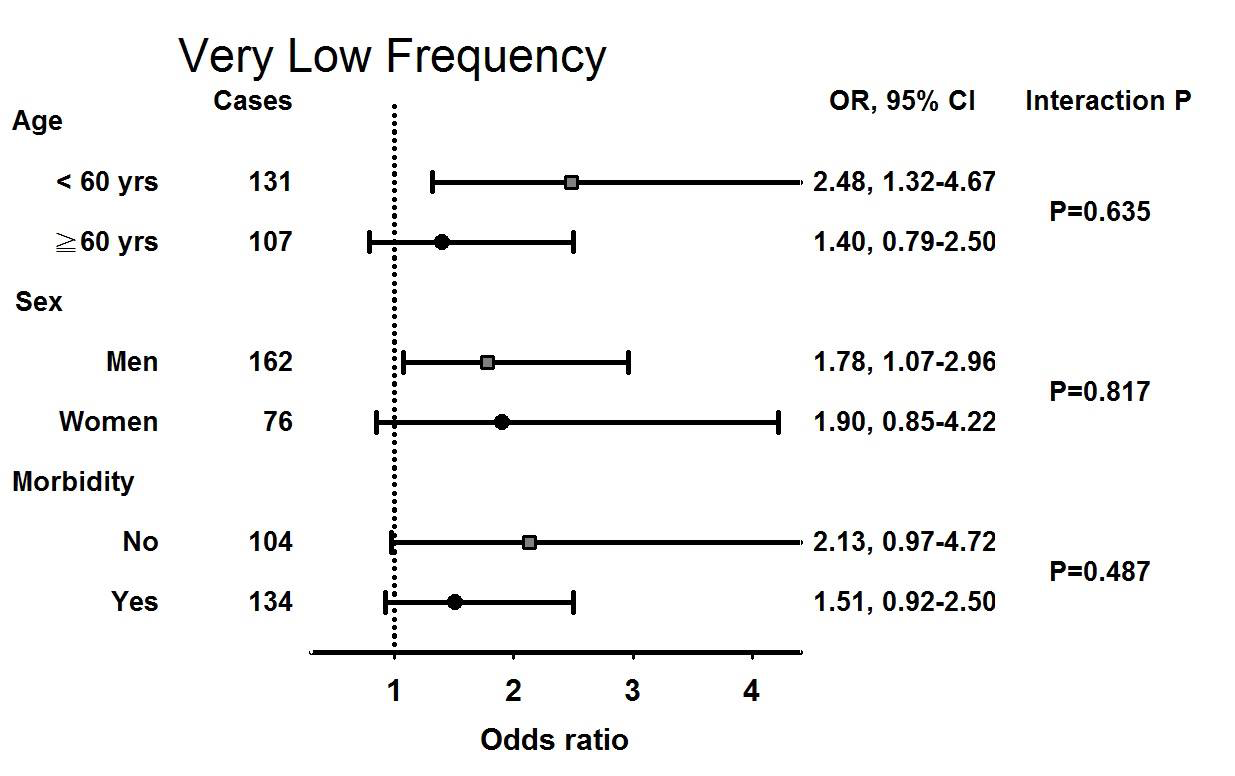

Supplement: S1 Fig — (TIF) [file pone.0144935.s001.tif]

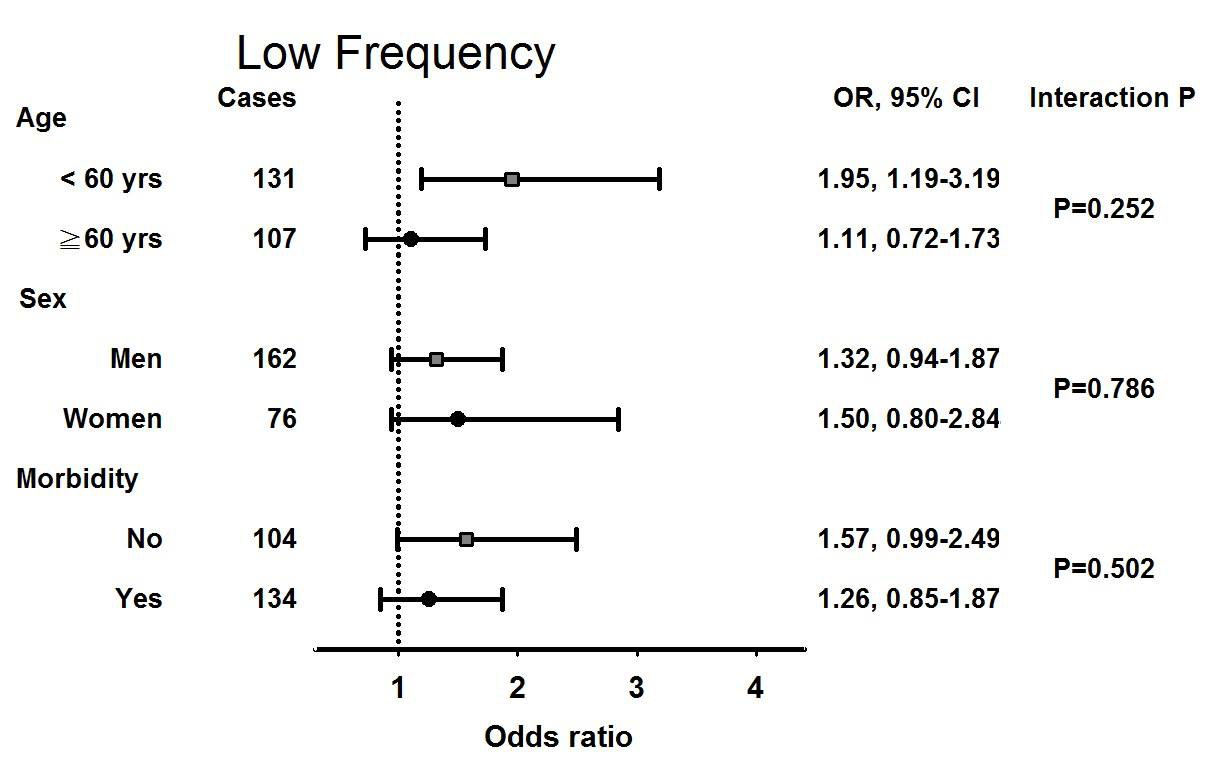

Supplement: S2 Fig — (TIF) [file pone.0144935.s002.tif]

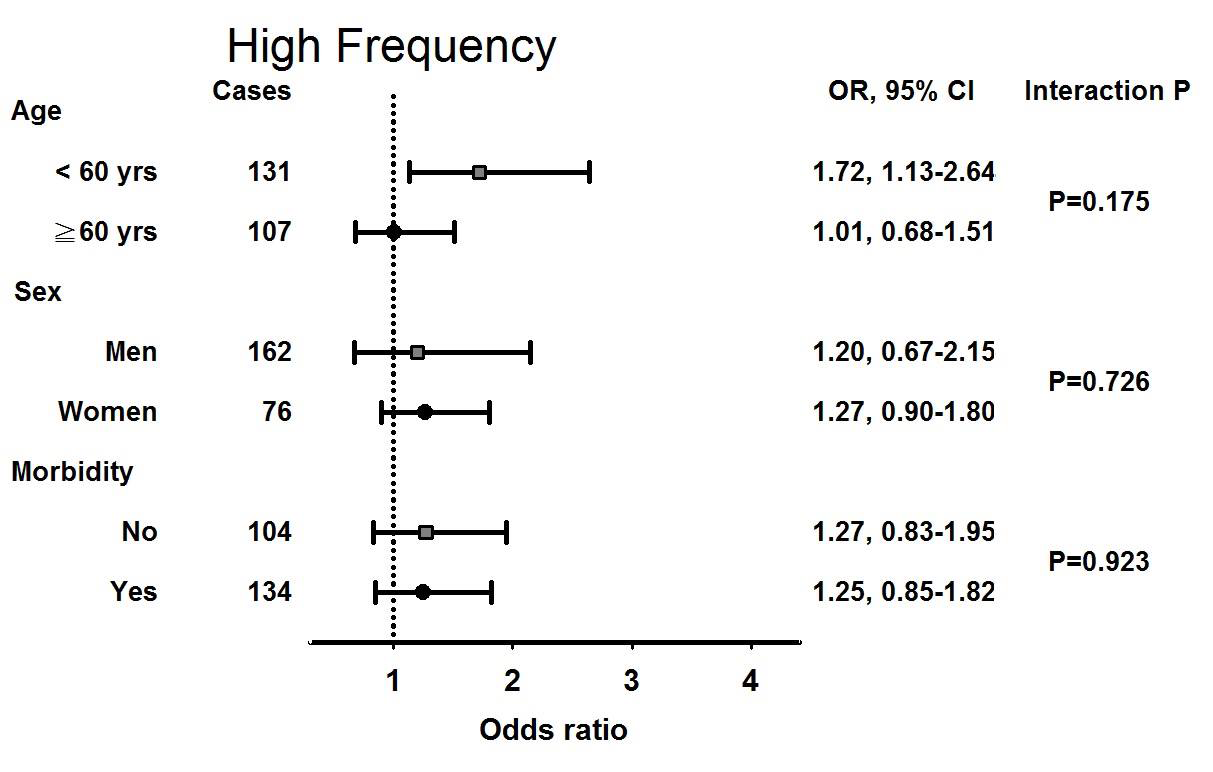

Supplement: S3 Fig — (TIF) [file pone.0144935.s003.tif]
